# Nosocomial Infections in Pediatric Population and Antibiotic Resistance of the Causative Organisms in North of Iran

**DOI:** 10.5812/ircmj.14562

**Published:** 2014-02-05

**Authors:** Salar Behzadnia, Alireza Davoudi, Mohammad Sadegh Rezai, Fatemeh Ahangarkani

**Affiliations:** 1 Antimicrobial Resistance Research Center, Department of Infectious Diseases, Mazandaran University of Medical Sciences, Sari, IR Iran; 2 Antimicrobial Resistant Nosocomial Infectious Research Center, Mazandaran University of Medical Sciences, Sari, IR Iran

**Keywords:** Infection, Children, Iran

## Abstract

**Background:**

Treatment of the nosocomial infections is complicated especially in children due to an increase in the antibiotic-resistant bacteria.

**Objectives:**

The aim of this study was to survey the nosocomial infections in children and determine the antibiotic susceptibility of their causative organisms in teaching hospitals in the north of Iran.

**Patients and Methods:**

The investigation was designed as a retrospective cross-sectional study. The study population consisted of patients under 12 years old, which were hospitalized in three teaching hospitals in the north of Iran and had symptoms of nosocomial infections in 2012. The required data of patients were extracted and entered in the information forms. The collected data were analyzed using SPSS (ver. 16). Descriptive statistics and Fisher’s exact tests (Monte Carlo) were used.

**Results:**

Out of the total number of 34556 hospitalized patients in three teaching hospitals, 61 (0.17%) patients were children under 12 years old age with nosocomial infection from which 50.81% were girls and 49.18% were boys. Most of these patients (55.73%) were admitted to the burn unit. The most common type of nosocomial infection (49.18%) was wound infection. Pseudomonas spp. (36.84%) and *Acinetobacter* spp. (28.02%) were the most common bacteria isolated from the clinical specimens. All the *Acinetobacter* spp. were multidrug-resistant. All the gram negative and gram positive bacterial species in our study showed high resistance to antibiotics.

**Conclusions:**

The rate of nosocomial infections was low in our study because the detection of nosocomial infection was based on the clinical grounds in most cases and laboratory reports might contain false-negative results. These results provide useful information for future large scale surveillance in the context of prevention programs.

## 1. Background

Nosocomial infections (NIs) remain a major problem in the health care centers across the world and leads to high mortality. NIs exist in a worldwide fashion. Globally, 8.7 % of the hospitalized patients are affected with NIs. These infections cause death, failure of surgeries, rejection of transplanted organs, failure of chemotherapies and increasing costs for patients and health centers, a longer stay in the hospital and mental and emotional stress (1-3). In Europe, the incidence of NIs in the general children ward is 1% and in the neonatal intensive care units have been reported to be 23.6 % (2). The most common type of NIs in children are bloodstream infections, pneumonia (ventilator-associated VAP), urinary tract infections (UTI), skin and surgical site infections (1). Organisms such as gram-negative bacilli, coagulase-negative staphylococci, coagulase-positive *staphylococci*, *pseudomonas* spp, and *streptococcus* are the main causes of NIs. A common problem in the treatment of NIs in pediatric wards in hospitals is increasing frequency of antibiotic-resistant organisms. Surveillance activities are the first step in developing infection control programs and may help in decreasing the incidence of infections and reducing costs (2). However there is a significant knowledge gap regarding the NIs due to the lack of enough data from the epidemiological studies: the reports coming from Iran are not enough and there has not been any report of surveillance of NIs in children from the north of Iran.

## 2. Objectives

The purpose of this study is survey of NIs and antibiotic susceptibility patterns of causative agents among the children admitted to the teaching hospitals affiliated with the Mazandaran University of Medical Sciences in order to help the physicians in choosing better antibiotics for the empiric therapy of these infections.

## 3. Patients and Methods

This was a cross sectional-retrospective study. The location of study was the general pediatric wards, NICU and burn wards of three teaching hospitals of the Mazandaran University of Medical Sciences (in north of Iran) including the Bu Ali Sina hospital, Shahid Zare Hospital and Razi hospital. This study was approved by the Ethics Committee of Mazandaran University of Medical Sciences (Code No: 9134, Date: July 11, 2012). Census method was performed for sampling. The study population included the children under 12 years old, hospitalized in these hospitals in 2012 who had symptoms of NIs. Infections (based on National Directory of Nosocomial Infections Surveillance System) (4), were defined as:

UTI: The patient must have at least one of the symptoms such as fever, dysuria, frequency, flank pain, suprapubic pain, nausea and vomiting plus positive urine culture or must at least have two symptoms such as fever, dysuria, frequency, flank pain, suprapubic pain, nausea and vomiting plus pyuria.

Wound Infection: Superficial surgical site infection is identified with at least one of the following characteristics: purulent discharge from the wound, organisms isolated from the fluid or superficial surgical tissue that should aseptic, at least one of the symptoms such as pain, swelling, redness or warmness, or diagnosis of the wound infection by the doctor. Respiratory Infection: Crackles on lung examination or radiographic findings plus at least one of the following: purulent sputum or positive blood culture or positive culture of the tracheal aspirate sample. Blood Infection: Blood culture grows a pathogenic organism, condition that is not related to the location of a localized infection or having fever, chills, decreasing blood pressure plus existing infections related to the skin in at least two blood culture samples (like diphtheroids, bacillus species, propionibacterium or coagulase negative staph).

Identification of the organisms causing infection were performed according to the standard microbiological procedures (5, 6). Antimicrobial susceptibility testing method, the disk diffusion (Kirby-Bauer) were performed according to the standard CLSI2010 (7). We gathered information from the demographic and clinical characteristics, risk factors, medical history, main diagnosis, type of NIs, type of the culture and entered them in the data forms. Then, the collected data were analyzed using SPSS software (ver. 16). Descriptive statistics and Fisher’s exact tests (Monte Carlo) were used for the statistical analysis.

## 4. Results

**Figure 1.**
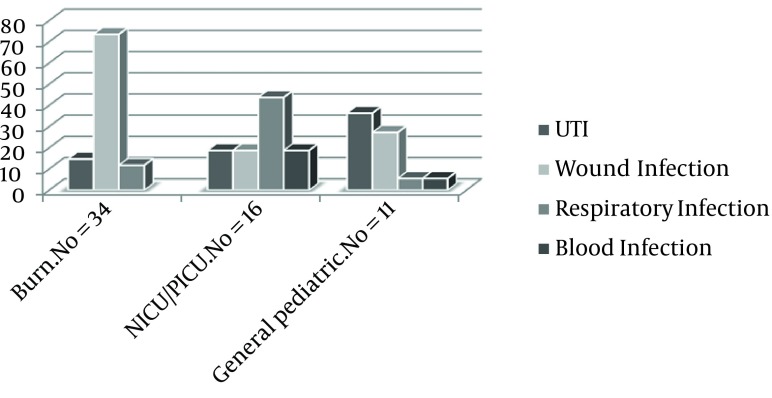
Distribution of Patients According to the Types of NIs by the Ward Type (P=0.273)

 From the total of 34556 hospitalized patients in three teaching hospitals, 61 (0.17%) patients were children under age 12 with NIs, from whom 31 were girls (50.81%) and 30 were boys (49.18%). The average age was 6 ± 4.32 (range 1 day, 12 year) years old. The average duration of hospitalization was 7 (range, 2-35) days. The most prevalent types of NIs were wound infection (50.81%, 95%:CI37.9-63.7), respiratory infection (21.31%, 95%CI: 10.7-31.8), UTI (19.67%, 95%CI: 9.4-29.9) and blood Infection (8.19%, 95% CI: 1.1-15.2), respectively. The prevalence of NIs in various wards is shown in Figure 1 and the demographic features, clinical characteristics and risk factors for each type of infection are described in the Table 1. The incidence of various causative organisms for NIs is listed in Table 2. Antibiotic sensitivity patterns of the bacteria that cause NIs are listed in Tables 3 and 4.

**Table 1.** Demographic Features, Clinical Characteristics and Risk Factors of Infection

	Wound Infection, No. (%)	Respiratory Infection, No. (%)	Urinary tract Infection, No. (%)	Blood Infection, No. (%)
**Gender**	-	-	-	-
Female	16 (51.6)	6 (46.2)	9 (75)	2 (40)
Male	15 (48.4)	7 (53.8)	3 (25)	3 (60)
**Age (year)**	7.09 ± 3.83	4.07 ±4.48	8.33 ±3.67	2.20± 3.03
**Average duration of hospitalization (day)**	7.06±5.5	8.07±1.9	5.6±2.5	7.6±3.6
**Risk factor**	-	-	-	-
Diabetes	3 (9.7)	-	-	-
HTN	4 (12.9)	-	-	-
Cardiovascular disease	3 (9.7)	-	-	-
Urine catheter	-	-	9 (75)	-
Steroid therapy	1 (3.2)	-	-	-
**Symptoms**	-	-	-	-
Fever	26 (83.87)	13 (100)	12 (98.4)	5 (100)
Dysuria	-	-	9 (75)	-
Frequent urination	-	-	9 (75)	-
Flank pain	-	-	7 (58.3)	-
Suprapubic pain	-	-	8 (66.7)	-
Nausea	-	-	-	-
Vomiting	-	-	-	-
Chest pain	-	-	-	-
Cough	-	10 (76.9)	-	-
Increase of sputum	-	5 (35.8)	-	-
dyspnea	-	7 (53.8)	-	-
Wound erythema	29 (93.5)	-	-	-
Wound oozing	24 (77.4)	-	-	-
Suture openings	21 (67.70)	-	-	-

**Table 2.** Causative Agents of Infections

	Wound Infection, No. (%) (no = 30)	Respiratory Infection, No. (%) (no = 13)	Urinary Tract Infection, No. (%) (no = 9)	Blood Infection, No. (%) (no = 5)	Total, No. (%) (no = 57)
***Pseudomonas.*spp**	12 (40)	4 (30.76)	3 (33.33)	2 (40)	21 (36.84)
***Acinetobacter.*** **spp**	10 (33.33)	6 (46.15)	0	0	16 (28.07)
***E. coli***	0	0	4 (44.44)	0	4 (7.01)
***C. freundii***	2 (6.66)	0	0	0	2 (3.50)
***Enterobacter.spp***	2 (6.66)	0	0	1 (20)	3 (5.26)
***Klebsiella.spp***	0	2 (15.38)	0	1 (20)	3 (5.26)
***S.Marcescens***	0	1 (7.69)	0	0	1 (1.75)
***S. aureus***	2 (6.66)	0	0	0	2 (3.50)
***S. saprophyticus***	2 (6.66)	0	0	1 (20)	3 (5.26)
***C.albicans***	0	0	2 (22.22)	0	2 (3.50)

**Table 3.** Antibiotic Susceptibility of Gram Negative Bacteria Isolated From Infection Sites

Antibiotics	*C.*F*reundii*, (%) (no = 2)		*S.Marcescens*, (%) (no = 1)		*E.coli*, (%) (no = 4)		*Klebsiella.spp*, (%) (no=3)		*Enterobacter. spp*, (%) (no = 3)		*Acinetobacter.spp*, (%) (no=16)		*Pseudomonas. spp*, (%) (no=21)	
	R^a^	I^a^	R	I	R	I	R	I	R	I	R	I	R	I
**Ceftriaxone**	-	100	-	-	100	-	-	100	-	33.3	100	-	94.4	5.6
**Ceftizoxime**	50	50	100	-	-	100	-	100	66.6	-	100	-	42.85	19.33
**Ceftazidime**	50	50	-	100	100	-	66.6	33.3	-	66.6	100	-	33.3	-
**Cefixime**	50	50	100	-	-	50	-	100	66.6	33.3	100	-	11.1	-
**Carbenicillin**	-	100	100	-	-	100	-	100	66.6	33.3	100	-	42.85	-
**Ampicillin**	100	-	100	-	100	-	100		66.6	33.3	100	-	57.14	4.7
**Ciprofloxacin**	50	50	-	-	100	-	-	100	100	-	100	-	94.4	5.6
**Norfloxacin**	-	100	-	-	100	-	-	100	100	-	100	-	42.8	-
**Nalidixic Acid**	100	-	-	100	100	-	66.6	33.3	-	100	100	-	23.8	-
**Gentamicin**	-	100	-	-	100	-	66.6	-	100	-	100	-	42.85	19.33
**Amikacin**	-	100	-	-	-	25	-	-	-	100	100	-	50	-
**Imipenem**	-	100	-	-	100	-	100	-	-	-	100	-	38.9	-
**Co – trimoxazole**	100	-	-	100	75	25	-	100	100	-	100	-	80.95	
**Tetracycline**	-	100	100	-	100	-	-	100	100	-	100	-	71.42	

^a^ Abbreviations: R: Resistant to antibiotics; I: Intermediate sensitivity to antibiotics

**Table 4.** Antibiotic Susceptibility of Gram Positive Bacteria Isolated From Infection Sites

antibiotics	*S. aureus*, (%) (no = 2)		*S. saprophyticus*, (%) (no = 3)	
	R^a^	I^a^	R	I
**Ampicillin**	100	-	100	-
**Carbenicillin**	100	-	33	-
**Penicillin**	100	-	100	-
**Oxacillin**	100	-	66	-
**Cefazolie**	100	-	100	-
**Ceftriaxone**	-	-	66	-
**Ceftizoxime**	-	50	100	-
**Ciprofloxacin**	100	-	100	-
**Vancomycin**	-	-	-	-
**Clindamycin**	-	50	-	-
**Erythromycin**	100	-	66	-
**Co - trimoxazole**	100	-	100	-
**Tetracycline**	100	-	66	-

^a^ Abbreviations: R: Resistant to antibiotics; I: Intermediate resistance to antibiotics

## 5. Discussion

The rate of NIs in our study was lower than other studies. The following points need to be considered. NIs detection was based on the clinical grounds in most of our cases; which raises the possibly of missing patients with subclinical infections and also might be due to the fact that laboratory reports might contain many false-negative results. Absence of facilities for culture of anaerobic bacteria in the north of Iran, low NIs reporting from wards, early discharge of the patients undergoing surgery, and lack of follow up in NIs patients referred to clinics, all can be a cause of falsely low reported rate of NIs. In this study, most of our patients were hospitalized in burn unit and most common type of NIs was wound infection which is consistent with earlier studies (8-10). The most common bacteria isolated from patients in this ward (burn unit) were *P. Aeruginosa* and *Acinetobacter* spp. The type of bacteria isolated from wound samples in Oncul et al. study was similar to our research (11). In Javanbakht et al. study in Mashhad, the highest frequency of cross infection was in burn ward and *Acinetobacter* spp. was the most frequent pathogen, which is different from our results. The high incidence of *Acinetobacter* spp. in their study may be due to the abundance of dry soil in Mashhad which is the origin of *Acinetobacter* (12). In the study of Coetzee et al. 44.81% of the isolated organisms from pediatric patients admitted to the burn unit was *P. Aeruginosa* which is similar to our findings (13). We observed that the most important risk factors in patients with wound infection were diabetes mellitus and use of steroids. The increased susceptibility to wound infection in diabetic patients is an established risk factor for NIs (14). Also, development of NIs was associated with the use of steroids in Rojas study (15).

We found that 26.20% of NIs patients were admitted to NICU/PICU and respiratory infection (43.75% of NIs cases) was the most common NIs in this ward followed by UTI, Blood infection and wound infection. Due to frequent airway suctioning, contamination of nurses' hands are major causes of respiratory NIs in this ward. In Raymond et al. study the rate of lower respiratory tract infections in PICU was 53%. Conversely, Pourakbari et al. reported the rate of respiratory tract infections to be 36% (2, 3). We noted that in our study there was no case of ventilator associated pneumonia was found; the fact that the ventilator was not used at all is one of reasons for the lower prevalence of respiratory infections in our study compared to other studies. Prevalence of NIs varies in different regions, for example, studies in the United States has shown that the incidence of NIs in NICU varied from 5.26 % to 12 %, but Abdel-Wahab et al. in Egypt, reported the incidence of NIs in the NICU to be 21.4% and in the Salamati et al. study prevalence of NIs was 40% (16-18). Comparing Salamati et al. results with our findings, the prevalence of NIs in NICU/PICU in our study was lower. Also, the types of NIs in their study were different from our results. We did not observe *S. aurues* in patients hospitalized in NICU/PICU in contrast to Salamati et al. study. In the general pediatric ward, the prevalence of NIs was 18.53% in our study. A total of 19.67% of patients in our study had UTI, from which 12.9% of patients with UTI were hospitalized in the general ward, which was consistent with reports of Pourakbari, Balat and Abdolioskouie (2, 3, 19, 20). The most common risk factor for UTI was urinary catheter (73.8%) consistent with Dashtbozorg et al. study (21). In our study we observed that the most causative agent of UTI was *E.coli*; according to many studies in Iran, the main cause of UTI is still *E. coli* (22, 23). We reported that 8.19 % (3 cases in NICU and 2 cases in General pediatric ward) of patients had nosocomial blood infection. These numbers were much lower than the results of Abdolioskouie (68.9%) and Becerra (18.1%) and are closer to the Pourakbari et al. findings (14%). The most common bacteria isolated from the blood infections in Pourakbari et al study were gram positive bacteria, but in our study it was *pseudomonas *spp (1, 3, 20).

In this study, most of the isolated bacteria were *Pseudomonas* spp and *Acinetobacter* spp. Also these bacteria have been reported as the most common cause of NIs in Hsueh and Ortega studies (24, 25). All *Acinetobacter* spp. isolated from clinical specimens were multidrug-resistant. Prevalence of the multi-drug resistant *Acinetobacter* spp. in countries of the Atlantic region have been reported to be 29.3% (26). Unfortunately treatment of the infectious diseases caused by *Acinetobacter* spp. is difficult because of the increase in the prevalence of multi-drug resistant strains (27-29). Death rate resulting from NIs caused by *Acinetobacter* spp. have been reported to be 7.8% to 23% (26). Between 58% to 96 % of *Acinetobacter* spp. which were the cause of NIs in Vahdani et al. study were multidrug-resistant (30). Pseudomonas species isolated from clinical samples in our study have shown different but still high level resistance to third-generation *cephalosporins*, aminoglycosides and *fluoroquinolones*. *Enterobacteriaceae* species in our study have been shown to have high level resistance to antibiotics. *E. coli*, *Enterobacter* spp., *Klebsiella* spp., *C. freundii*, and *S. marcescens* have been shown to have 50% to 100% resistance rate to antibiotics. In Bean et al. study, the rate of resistance of *E. coli* to ampicillin, gentamicin, cotrimoxazole and ciprofloxacin, were lower than our results (31). It seems that, the high incidence of antibiotic resistance to *Enterobacteriaceae* in our study is due to the low number of these organisms compared to Bean et al study. In our study, resistance to amikacin was low in gram negative bacteria except *Acinetobacter* spp. and *Pseudomonas* spp.; *C. freundi*(100%), *E.coli *(25 %) and E*ntrobacter.* spp (100%) were sensitive to amikacin.

*S. aurous* and *S. saprophyticus* had very high rate of antibiotic resistance. In the study of Molaabbaszadeh et al. *S. aureus* resistance was low for ciprofloxacin, clindamycin, and cotrimoxazole (32). In our study, all samples had (100%) resistance to these antibiotics; however among *S. aureus* and *S. saprophyticus* organisms resistance to vancomycin were not observed. In Higashide study, 100% of *S. saprophyticus* isolates were resistant to oxacillin, but in our study rate of oxacillin resistance was lower (33).The total rate of NIs was low in our study. Diagnosing NIs in our hospitals was mainly by physicians and according to the clinical criteria for reporting NIs, so the rate of inappropriate administration of antibiotics was very high. Therefore, microbiological findings might not have been valid because empirical treatment had already been started before obtaining the samples from patients. Also, our laboratory findings are not accurate and contain many false negatives. Before starting the empiric treatment or/and antibiotic prescription, physicians need to take into the consideration the prevalence of NIs and antibiotic resistance patterns of the bacteria (isolated from clinical specimens, air and equipments) in different wards.
